# Rural Population Aging and the Hospital Utilization in Cities: The Rise of Medical Tourism in China

**DOI:** 10.3390/ijerph17134790

**Published:** 2020-07-03

**Authors:** Bing Hu, Daiyan Peng, Yuedong Zhang, Jiyu Yu

**Affiliations:** 1 Department of Economics, Huazhong University of Science and Technology, Wuhan 430074, China; academicpeng@126.com; 2 Research Office of E’zhou People’s Government, E’zhou 436000, China; yuedong2017@126.com; 3 School of Finance, Hubei University of Economics, Wuhan 430205, China; jiyu_yu@hbue.edu.cn

**Keywords:** rural population aging, medical tourism, inpatient visits, rural urban disparities

## Abstract

The disparity of rural and urban hospital utilization has aroused much concern. With the improvement of their living standards, patients in rural areas have an emerging need for traveling across borders for better medical treatment in China. This paper reveals the medical tourism of rural residents towards urban hospitals driven by hospital needs and points out that such disparities may be caused by medical tourism. The ratio of people aged 65 and above in total rural populations was used to identify the potential target customers for medical tourism. Based on rural and urban datasets ranging from 2007–2017 on the provincial level, this paper presents a mobile treatment model and market concentration model with an ecological foundation. The feasible generalized least squared approach was used in the estimation of the fixed-effect regressions. The study found that there was a positive and significant relationship between rural old-age ratios and urban inpatient visits from different income groups. On average, a one percent rise in rural old-age ratio would increase the inpatient visits of urban hospitals by 138 thousand persons. There was also a positive and significant relationship between the rural old-age ratio and the market concentration of urban inpatient visits. It was found that the rural old-age ratio significantly influenced the market concentration of urban inpatient visits in the middle-high income regions. The research showed that each income group from the rural aged population had participated in medical tourism, traveled to urbanized regions and made inpatient visits to urbanized medical facilities. It was also indicated that the rural aged population, especially from the middle-high income groups had a positive and significant influence on the market concentration of urban inpatient visits in the province.

## 1. Introduction

For a long time, large geographic disparities of urban rural healthcare utilization have attracted much attention. The rural–urban variations of healthcare utilization have often been linked with the shortage of fairness and medical opportunities of rural communities due to the availability of medical limited facilities [1,2,3]. However, the study from Finkelstein, Gentzkow and Williams (2016) found that 40% of geographic variances resulted from the demand side, including the medical choices of the patients [4]. Hence the phenomenon of medical tourism should not be ignored. Through the analysis of the Chinese context which our study emerging from, this paper proposes that such variations could be caused by another reason: the mobility of rural patients for medical purposes. This paper argues that a rural agent may relocate for a medical purpose from a native town to a city of the same province in the Chinese context. An interesting economic assumption puts that human is rational and must maximize its benefit [5]. Existing literature supports that people conduct cross border treatment for better quality service or cheaper medical prices at certain budget constraints [6,7,8]. However, the patient mobility phenomenon between rural and urban areas have not been studied yet in the Chinese context.

This study was carried out in China. A large scale of urbanization and dramatic social and economic changes in rural areas lead to the advent of medical tourism by rural dwellers. Typically, the medical system in China is similar to quasi-public goods. Both rural and urban medical systems are composed of primary care institutions and higher-grade hospitals. However, rural hospitals often lag behind urban hospitals in state-of-the-art medical technology and equipment. Even for the low socioeconomic status rural dwellers, the technology level for rural hospitals sometimes could not satisfied their needs [9].

This study aims to identify the medical tourism of the rural population from the aggregate data. In the ecological approach, one uses data that is aggregated by character units to investigate the behavior of the individuals comprising those units with aggregate level regression [10]. When rural dwellers are classified into a group with homogeneous hospital needs, an ecological approach was implemented to allow the causal inference of decision making from individual to aggregate level [11]. From the contribution of methodology, the ecological approach was an important technique to deal with the medical tourism of rural patients by geographic and age units. Empirical models were established based on the ecological foundation of individual-level medical choice, making it more rigorous to infer the cause-and-effect relationship between the medical tourism of rural population and the urban inpatient visits on aggregate level [12]. From the literature part, the study gave empirical evidence of medical tourism in the Chinese context and shed light on the other developing countries with similar medical settings.

### 1.1. The Medical System in Rural and Urban China

**Figure 1:**
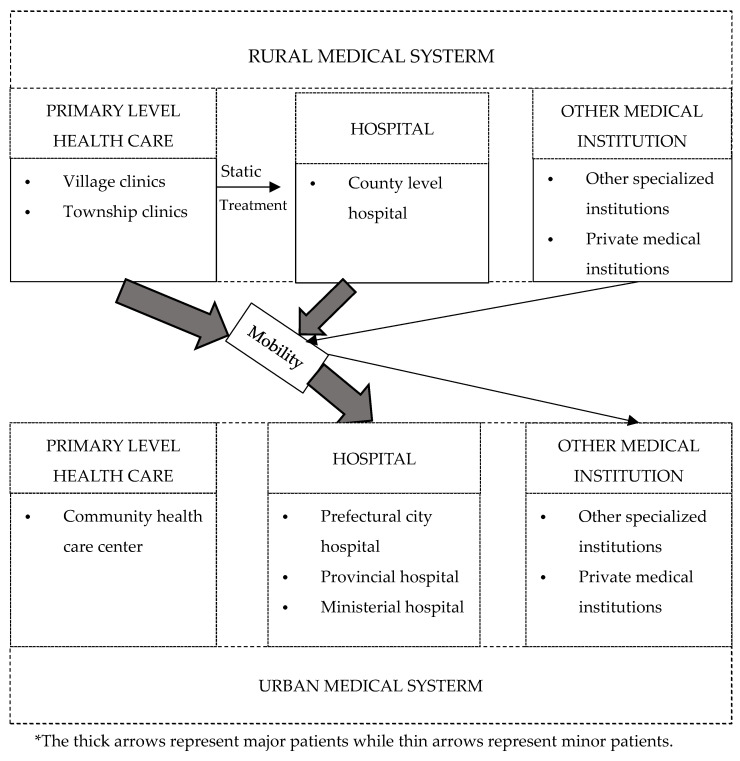
Medical systems in China.

The mobility of rural patients is closely driven by the institutional characters of the Chinese medical system. The medical system of rural and urban counterparts is represented in Figure 1. The rural medical system consists of primary level health care institutions, county-level hospitals and other medical institutions. Primary level institutions are the lowest unit in the medical system and aim to serve the basic medical needs of the residents. In rural areas, primary level care includes village and township clinics. The rural medical system of China often emphasizes primary level medical services, leaving rural residents limited choices. Despite opportunities for rural patients to be transferred to higher-level hospitals in the local system, many rural residents would still need to travel to the leading urban hospitals for better treatment. Hospitals in urbanized municipal districts are frequently higher grades and medical quality compared to rural hospitals. Restricted to technique and staff in county-level hospitals, urban hospitals are more attractive to rural patients. According to the provincial income per capita data from China Statistical Yearbook from 2007 to 2017, the average growth rate of rural income is 1.4 percent higher than that of the urban population. With the rising of rural income, primary level care is not sufficient for rural medical demand [13]. Hence the medical tourism phenomenon should be noticed and further discussed in the literature.

### 1.2. Aging and Healthcare Utilization

Anderson and Newman (1973) first proposed the health care utilization model, including age as a predetermined factor for healthcare demand [14]. Many studies proved that the aged population had a significant impact on healthcare utilization from aggregate panel data. It has been found that the healthcare expenditure of the aged population has been 2–6 times higher than younger groups [15,16]. The probability of health care utilization was also the highest among people above 65 based on aggregate panel data [17,18]. Several studies used age as a proxy for healthcare demand in the aggregate level. Palangkaraya and Yong (2009) used age to predict healthcare demand on aggregate level [19]. Old-age ratio is defined as the ratio of population aged 65 and above in total population. Gaynor and Anderson (1995) used the old-age ratio as a proxy for the demand-side factors of hospital bed utilization [20]. In addition, evidence from individual-level surveys illustrates the positive relationship between the old age group and healthcare utilization. Aging is demonstrated to be strongly linked with chronic diseases and morbidity in a society [21,22,23]. Aging changes the functional ability of the body largely and is an important predictor for healthcare needs [24,25,26]. However, some researchers argue that not only age, but also the time before death determined the amount of health care utilization [27,28]. Generally, the old age population is the main driving force for hospital utilizations. However, how the age influences medical tourism in rural and urban China would be in further discussion.

### 1.3. Medical Tourism with an Ecological Approach

Medical tourism is a phenomenon arousing concern recently in health economic literature. Part of the literature discussed the mobility of patients from supply side factors. It was found that people would like to conduct cross border travels for lower prices, easier accessibility or better quality in medical care [6,29,30]. Eom, Yu and Han (2019) based on 729 papers about medical tourism in Korea found that the growth of the emerging markets, the dominated medical competitiveness, and the legitimate price in Korean clinics would help to contribute to the growth of international medical tourists to Korea [31]. Several studies also found the patient-centered factors, such as race, demographics, socioeconomic status, etc. have a significant influence on their medical choices [32,33,34]. Finkelstein, Gentzkow and Williams (2016) investigated the influence of individual medical choice and found the rise of medical tourism along with socioeconomic factors would bring about over 40% geographic variations [4]. Han and Hwang (2018) found that traveling abroad for a medical purpose not only influenced by the product quality and cost of medical services in the destination country, but also by travelers’ experience and satisfaction [35]. Medical tourism studies in the Chinese context are very limited. There was a qualitative study earlier about the motivation for medical tourists from China mainland to Hong Kong [36]. Recently, another study from Cai, Liu and Tao (2018) found the higher grade of the hospital attracted the patient from undeveloped areas. However, this study mainly focused on hospital performance and efficiency evaluation, not concentrated on medical tourism domestically [13].

In this paper, we identify the medical tourism of the rural population from rural to urban China with the aggregate level data. To solve this problem, we adopt an ecological approach to infer individual behavior from aggregate data. In the ecological approach, regression units are made up of the characteristics of individuals in aggregate level [37]. It was therefore proposed that aggregate data can be valuable allies to the sample surveys if the aggregate data studies carefully and thoroughly the recurring patterns of the target units and the characters of outcome variables over time [38,39]. The ecological approach is initially used to predict the proportion of each candidate’s votes in electoral districts through the voting patterns of the electorates with different demographic groups [40,41]. Blalock first gave two adequate assumptions for the ecological regression if the correlation in aggregation regression can represent the relevant parts in individual regression: 1. The coefficient regression for $\bar{X},\bar{Y}$ among aggregate units would be equal to the coefficient regression for X, Y among individuals if individuals are grouped in a way related to their scores on X; 2. $\bar{X}$ and error term ε are independent with $\bar{Y}$ in aggregation regression [42]. Blalock ‘s approach is only restricted when X and Y are intervals, while Goodman extended it into nominal and ordinal variables. Goodman’s approach was followed when there was a probability event happening for the aggregate unit of individuals. The coefficient regression was calculated by the percentage of X from category I and the percentage of Y from category M [43].

The study is contributed to the literature in two parts: first, it revealed the medical tourism of the rural population in the Chinese context and helped to explain the rural urban geographic variations in health care. Second, the ecological foundation was incorporated into the healthcare utilization model, making it more rigorous to conduct causal inference from individual behavior.

## 2. Methods

### 2.1. Study Design

**Figure 2:**
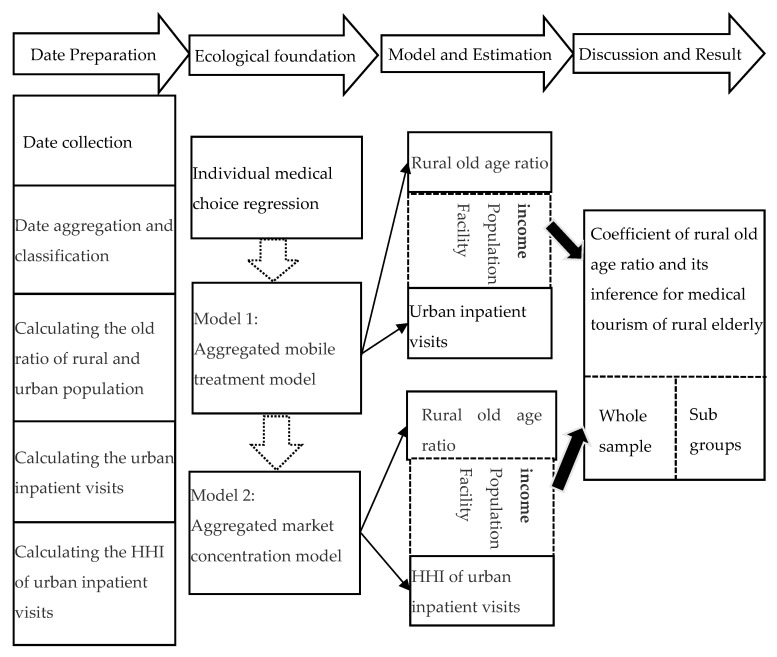
Conceptual scheme of the study design.

In this study, we used secondary databases—including demographic database of rural and urban dwellers respectively and the hospital utilization database of urban hospitals—on the provincial level. Initially, we had a total of 341 observations from 2007–2017. Due to lack of rural data, Beijing and Shanghai province were deleted and a total of 319 observations were selected. The aggregate models were built on the individual medical choice model through an ecological approach. The steps of this study are presented in Figure 2. First, we aggregated the data on rural and urban divisions of a province. Second, we calculated the age distribution of the total population by rural and urban regions in a province. Third, we calculated the inpatient visits by rural and urban hospitals and derive the inpatient visits of the urban primary level above hospitals. Fourthly, we derived a mobile treatment model based on the ecological analysis. The mobility of rural elderly can be identified through the coefficient of rural old-age ratio towards urban inpatient visits. Fifthly, we constructed the market concentration index of the urban inpatient visits and established a market concentration regression model. The medical tourism of rural elderly can be identified through the correlation between rural old-age ratio and the market concentration of urban inpatient visits. In the end, we discussed the regression results and made a conclusion.

### 2.2. Data Source

The data source ranged from 2007 to 2017 on an aggregate level, including rural and urban data of total 29 provinces, 319 observations. Shanghai and Beijing were excluded in the sample because, after 2016 county areas of both provinces were merged into municipal districts. Inpatient visits to urban hospitals and medical institutions were aggregated data by province from China Health Statistical Yearbook. It was hospital discharge data summarized by geographic units. According to the classification criteria for rural and urban bed supply, we defined the county and the below regions as rural areas and the prefecture-level city as urban areas. Based on the criteria above, the data of urban hospital inpatient visits were obtained by the provincial total inpatient visits minus rural total inpatient visits and minus urban primary care level inpatient visits [44]. The classification of hospitals and primary care institutions is shown in Figure 1 based on China Health statistical yearbook. The data above was double-checked and linked with hospitalization service entities of medical institutions on primary level and hospital level, respectively from China Health Statistical Yearbook.

HHI of urban inpatient visits in a province was based on the value of urban and total inpatient visits of hospitals and medical institutions of each province. The population old ratio was available by village, town and city base from China Population 1% sampling survey, China Population & Employment Statistics Yearbook. Then we calculated the old dependency ratio of the rural and urban areas of each province manually.

Population data were calculated on urban and rural categories on the provincial level. The urban municipal population was obtained from China Urban-Rural Construction Statistical Yearbook. The provincial population was obtained from the China Statistical Yearbook. The per capita income of rural and urban residents was obtained from the China Statistical Yearbook. It is the aggregate data based on the weighted mean of a household survey of each province. Rural water improvement, the number of beds per hundred persons were obtained from the China Statistical Yearbook. Urban road area per capita and urban road length were obtained from China Urban-Rural Construction Statistical Yearbook.

### 2.3. Variables

The two outcome variables of this study were selected: inpatient visits for urban hospitals, including hospitals and other medical institutions above primary care level referred to Figure 1; the Herfindahl Hirschman Index (HHI) of urban inpatient visits by province. Inpatient visits of urban hospitals were equal to the total inpatient visits of urban hospitals and medical institutions minus the urban primary level inpatient visits. The primary level institution is to serve urban residents within the communities; therefore, it is not available for rural patients. The HHI of urban inpatient visits measured the market concentration of urban hospital inpatient visits in a province. It is calculated by the squared of inpatient visits of urban hospitals divided by the squared of aggregate inpatient visits of a province [45].

The key explanatory variable was the rural old-age ratio, which measured the proportion of people above 65 in total population in rural areas [16,18,19]. In addition, the demand factors of the rural population were controlled by rural population size, income per capita of the rural population. The demand factors of the urban population were controlled by urban population size, urban old-age ratio and urban income per capita. The weight of the urban population in the province was controlled by the HHI of the urban population in the market concentration model. The facility factors contained the social structure and environmental variables for urban inpatient visits. The number of beds per hundred persons in rural areas measured the facility of rural hospitals and would improve the competition between rural and urban hospitals [46,47]. The ratio of rural water improvement helped to improve the average health condition of the rural population and decrease their medical demand [48,49]. The infrastructure variables would influence the accessibility and traffic convenience of potential customers for medical seeking. Hence urban road square per capita, urban total road length would be included.

### 2.4. Ecological Foundation

**Figure 3:**
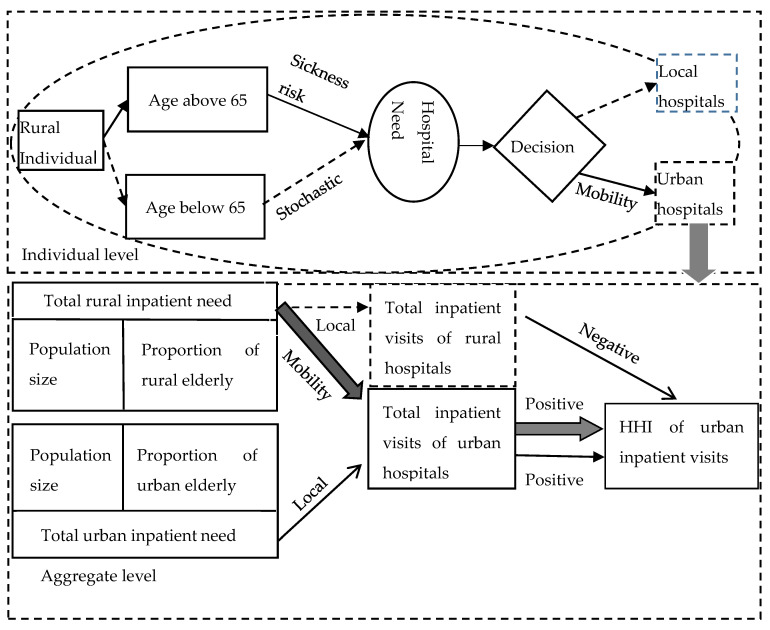
Ecological foundation of the aggregate level medical choice model.

The medical choice models from an individual level to aggregate level are presented in Figure 3. In the ecological approach, regression units are made up of the characteristics of individuals in aggregate level [10]. Hence, the ecological foundation of the medical choice model on the aggregate level was established on how an individual unit clustered into a group [11]. In the individual-level model, there are two prerequisites of medical tourism to be noticed: first, there is long-lasting hospital need for individuals in an area, so the medical tourism is meaningful and significant to be discussed; second, the destination of medical treatment should be different from the location of residence. Literature shows that hospital utilization of the aged population was 2–6 times higher than younger groups [15,16,17]. Hence, the rural people below 65 have scholastic hospital need and the elderly people have stable hospital needs, increased by age. Medical tourism of individual can be identified from the probability of personal urban inpatient visit increased with aging.

The aggregate level models were based on the characters of medical tourism at the individual level. Total urban inpatient visits included both urban and rural patients. Hence, we controlled both urban and rural hospital demands. A set of unobserved demographic factors such as education, health habit, rate of infectious diseases, etc., were captured by the coefficient of population and reflected the influence of demographic factors other than age on the aggregate level model. The destination of medical treatment was set as urban areas. Old age population in rural areas was the target group for the study compared with the younger population. In this study, without knowing the exact percentage of rural patients from urban inpatient visits—we used the rural population above 65 into a unit to proxy those rural dwellers who had significant hospital needs [39]. Hence, the medical tourism of rural elderly would be identified from the regression coefficient of rural old-age ratio related to urban inpatient visits at the aggregate level [42,43].

Further, we can identify the medical tourism of the rural population with the changes in the market concentration of urban hospital inpatient visits to secure cause-and-effect relationship. The logic behind lay that: If the rural elderly decided traveling for urban inpatient services when he had hospital needs, then this mobile visit would add the concentration of inpatient visits from urban hospitals and reduce the concentration of inpatient visits from rural hospitals at the same time [47,48]. Hence medical tourism of rural elderly would be further identified from the regression coefficient of rural old-age ratio related to HHI of urban hospital inpatient visits at the aggregate level.

### 2.5. Mobile Treatment Model

Based on the ecological foundation of the medical choice models in Figure 3, this article presents the medical tourism of rural population to urban hospitals in the mobile treatment model. The hospital needs of the rural population were captured by a group of people above age 65 based on the ecological foundation [39,42,43]. Other factors related to hospital utilization were based on the extension of Anderson’s behavior model [14]:

(1) $$y_{it}=\alpha +\beta_{1}Rold{\;}_{it-1}+\beta_{2}Rpop{\;}_{it-1}+\beta_{3}Rincome{\;}_{it}+\theta_{1}Uold{\;}_{it-1}\;\;\;+\theta_{2}Upop{\;}_{it-1}++\theta_{3}Uincome_{it}+\delta Facility_{it}+\lambda_{t}+\mu_{i}+\varepsilon_{it}$$

$y_{it}$ is the outcome variable, representing the inpatient visits for urban hospitals and other medical institutions above the primary care level (seeing Figure 1), aggregated on province i, year t. The outcome variables, along with rural and urban population size were measured by 10,000 persons per unit and recorded as a decimal. Hence the Poisson regression would also not suit well. In this case, we treated all variables as interval and the model was estimated by Feasible Generalized Least Squares method to reduce heteroscedastic disturbance.$\;Rold_{it-1}$ is the key explanatory variable measuring the old ratio of the rural population in the previous phase [19,50]. $\beta_{1}$ measures the influence of rural old-age ratio towards inpatient visits of the urban hospitals and is the key interesting coefficient for the study. $\beta_{2}$ measures the influence of rural population size in the previous phase.$\;\beta_{3}$ measures the influence of income per capita of the rural population. $\theta_{1}$ measures the influence of the old ratio of the urban population in the previous phase.$\;\theta_{2}$ measures the influence of urban population size in the previous phase. $\theta_{3}\;$measures the influence of income per capita of the urban population. δ measures the influence of facility factors, including numbers of beds per hundred rural persons, benefit ratio of water improvement for the rural population and urban total road length. $\lambda_{t}$ is the time effect,$\;\mu_{i\;}$is the fixed effect on the provincial level,$\;\varepsilon_{it}\;$is the random error term.

According to literature, the level of income influences the realization of medical needs [51,52]. We divided the sample into the low, middle, high-income groups based on the ranking of real per capita income of rural population based on the year of 2007. If the per capita income of the rural population falls within the bottom 20%, it is classified as a low-income group. If the income per capita of the rural population falls within 20–80, it is classified as the middle-income group. If the income per capita of the rural population falls within the top 20%, it is classified as the high-income group. Hence, we further exam the coefficient of the rural old-age ratio under different income groups based on model (1).

### 2.6. Market Concentration Model

If there were rural dwellers seeking inpatient services to urban hospitals, the demand of the rural aged population should contribute to the market concentration of urban inpatient visits within a province [53,54]. We use market concentration model to further exam the existence of rural medical tourism as below:

(2) $${\mathrm{HHI}}_{it}=a+\gamma HHIpop_{it-1}+\beta_{1}Rold_{it-1}+\beta_{2}Rincome{\;}_{it}+\theta_{1}Uold_{it-1}\;+\theta_{2}Uincome_{it}+\delta Facility_{it}+\lambda_{t}+\mu_{i}+\varepsilon_{it}$$

The model is estimated by the Feasible Generalized Least Squares method. ${\mathrm{HHI}}_{it}$ is the outcome variable representing the concentration of inpatient visits to urban hospitals and other medical institutions. Following Deng and Pan (2019), it is calculated by the squared of inpatient visits of urban hospitals divided by the squared of aggregated inpatient visits of a province [45]. $Rold_{it-1}$ is the key explanatory variable measuring the old ratio of the rural population in the previous phase. $\beta_{1}$ is the key interested coefficient to be estimated, which measures the influence of the old-age ratio of the rural population on the market concentration of urban inpatient visits above the primary care level. γ measures the influence of the urban population concentration in the previous phase. $\beta_{2}\;$measures the influence of income per capita of the rural population. $\theta_{1}$ measures the influence of the old ratio of the urban population in the previous phase. $\theta_{2}\;$measures the influence of income per capita of the urban population. δ measures the influence of facility factors, including numbers of rural beds per hundred persons, benefit ratio of water improvement for the rural population and average urban road per capita. $\lambda_{t}$ is the time effect for year $t\;,\;\mu_{i\;}$ is the fixed effect on the provincial level;$\;\varepsilon_{it}$ is the random error term. We further exam the coefficient of rural old-age ratio under different income groups in the same criteria with the model (1).

### 2.7. Availability of Data and Materials

The dataset analyzed in the study is from open publications and can be got on request. The source of data was described in the reference.

### 2.8. Ethics Approval and Consent to Participate

Approval for the study was granted by the academic advisory board of the Department of Economics, Huazhong University of Science and Technology, China. The data are obtained from the publication and yearbooks. Hence it is exempt from the approval submission procedure towards relevant parties. A written consent was signed with each participating author.

## 3. Results

### 3.1. Descriptive Statistics

Table 1 indicates the descriptive statistics of the variables in the study. Except for Beijing, Shanghai, there are 319 observations in the sample. Overall, the mean of total urban inpatients was 2.424 million. For the bottom 20% of low-income regions, the urban inpatient visits are also the lowest among the group (1.111 million). It is nearly half of the urban inpatient visits in the middle-income group and nearly 1/3 in the high-income group. The average HHI of urban hospital inpatient visits is 0.22, representing the concentration of urban hospital inpatient visits within the province. It is highly concentrated in the groups of middle and high income.

The mean of rural old-age ratio was 9.36% overall. For low-income groups, the ratio is 7.82% on the average, lower than those of middle and high-income groups. The mean of urban old-age ratio is 8.79% on the average. The mean of urban old-age ratio is reduced in the high-income group.

The mean of the rural population is 31.84 million people, including all regions below the county level. The mean of the urban population is 12.87 million people and it is increased in the high-income group. The mean of rural per capita income is 6,795.53 Chinese Yuan. The mean of urban per capita income is 19,000 Chinese Yuan. Both rural and urban income was adjusted with the CPI index based on 2007. The average HHI of the urban population is 0.09 and the higher scores on HHI represent a higher concentration of urban population within a province. The number of beds per 100 persons is 0.3 in rural areas on average. Over 72.76% of rural residents have benefited from water improvement. The mean of urban road per capita is 14.63 square meters per person. The mean of urban road length is 11,000 kilometers.

### 3.2. Mobile Treatment Regression

Table 2 illustrates the regression results about the relationship between the rural old-age ratio and inpatient visits of urban hospitals based on model (1). The result from column (1) showed that one percent increase in rural old-age ratio in the previous phase would improve the inpatient visits of urban hospitals by 138,646 persons (*p* < 0.001). The coefficient of rural old-age ratio was positive and significant in different income regions (*p* < 0.05). The coefficient of rural old-age ratio in high-income regions was larger than those in low and middle- income regions. It showed that the rural aged population of the high-income region had more medical travels towards urban hospitals than the people in low and middle- income regions.

Table 2 also indicates that the rural population size was significantly and positively related to inpatient visits of urban hospitals in the whole sample (*p* < 0.05). The coefficient of rural population size measures the influence of rural demographic factors after adjustment age-disease risks. It was negative and insignificant in the low-income regions, negative in the middle-income regions and positive in the high-income regions. It means that the increased numbers of the total rural population, especially for the working-age population, would reduce medical tourism of rural population in the low and middle- income regions. However, the increase of population size would increase the medical tourism of rural population from the high-income regions. This can be explained by the fact that the rural people below 65 have restricted hospital needs in the low and middle-income regions.

Table 2 illustrates the result that the urban old-age ratio was insignificant related to inpatient visits of urban hospitals in the whole sample. The coefficient of urban old-age ratio was positive in the low-income regions and turned negatively significant in the high-income regions. There was a possible explanation for the negative coefficient of urban old-age ratio: the population aging not necessarily caused inpatient visits when it contained information about the healthier and longer life expectancy of individuals. With the improvement of living standards and life expectancy, the hospital need of the urban aged population may decrease and chronic diseases increased [55,56]. In this case, literature shows that more chronic diseases are spreading among the working-age population [21,22,23]. The overall positive and significant coefficient of urban population size supported the above inference. The coefficient of urban population size was insignificant in low-income regions, but positive and significant in middle and high-income regions. It means that the numbers of the urban population, especially for the working-age population, were positive and significant to inpatient visits of urban hospitals in middle and high-income regions.

The result from Table 2 column (1) shows that, on average, both rural and urban per capita income was not related to the inpatient visits of urban hospitals. The increase in income per capita reduced the medical tourism of the rural population in the high-income regions, but it did not influence hospital demand of the urban population overall. The coefficient of per capita income was not consistent among subgroups for urban and rural, respectively. This can be explained by the dual effects of income on healthcare utilization which refers to Grossman. The first effect of income is to release the hospital demand with the increasing budget level. The second effect of income is health promotion; hence, it would reduce the probability of sickness and decrease hospital demand [57,58].

The ratio of the rural population that benefits from water improvement was significant and negatively related to the inpatient visit of urban hospitals in the whole sample and all subgroups from Table 2 (*p* < 0.05). The coefficient of the ratio of the rural population benefitting from water improvement was the highest in high-income regions. It means that the rising coverage of rural purified water has the greatest influence on the medical tourism of the rural population in high-income regions. The coefficient of urban total road length was significantly positively related to inpatient visits of urban hospitals in the whole sample and lower-income regions (*p* < 0.01), but not significant in high-income regions. It means that the improvement of infrastructure has a more significant influence on the inpatient visits of less developed areas. On average, there was no significant relationship between the rural bed supply and the inpatient visits to urban hospitals. The coefficient of rural beds was negative and significant only in high-income regions, but not in low and middle-income regions. It could probably be explained that due to the lower technological level, even the rural beds rose in low and middle-income regions, the rural population still needs better inpatient services in the cities.

### 3.3. Market Concentration Model Regression

Table 3 illustrates the results from the relationship between the rural old-age ratio and the concentration of urban inpatient visits based on model (2). From column (1) one percentage raise of the rural old-age ratio would increase the HHI of the urban inpatient visits by 0.007 in the whole sample (*p* < 0.01). The coefficient of the rural old-age ratio was positive and significant in the middle high-income regions (*p* < 0.05), yet it had no statistical influence on the low-income regions. It means that the rural elderly from low-income regions probably visited rural hospitals more than urban hospitals. However, the rural elderly from middle-high income regions had significant medical tourism and contributed to the market share of the inpatient visits of urban hospitals in the whole province.

On one hand, Table 3 illustrates the results that the increase in urban old-age ratio was significantly and negatively related to the market share of urban hospital inpatient visits in the whole sample (*p* < 0.01). It means that the inpatient visits to the urban hospitals were not concentrated among the urban old population. The negative coefficient of urban old-age ratio probably due to the level of hospitals in our study is restricted to the primary level above hospitals. On the other hand, with the improvement of living standards, the urban elderly live longer with more chronic diseases [21,22,55]. Hence their medical need may be shifted from inpatient need to primary level care and outpatient services.

Table 3 column (1) illustrates the coefficient of the income per capita of rural and urban population is approaching zero. It means the rural and urban income has little influence on the concentration of urban inpatient visits. The coefficient of HHI of the urban population was positive and significantly related to the concentration of urban inpatient visits from the whole sample and subgroups. It means the concentration of the urban population is a driving factor for the concentration of urban inpatient visits. The coefficient of urban road per capita was positive yet insignificant to the HHI of urban inpatient visits in the whole sample. It was positive and significant in the low and middle- income regions and insignificant in the high-income regions. This means that the urban road per capita promoted the market concentration of urban inpatient visits in less developed regions, while it had an insignificant influence on high-income regions. The coefficient of the ratio of the rural population benefit from water improvement was negative and significantly related to the HHI of urban inpatient visits in the whole sample, but not very significant in subgroup areas. The coefficient of the beds per hundred rural persons was negative and significantly related to the HHI of urban inpatient visits in the whole sample and lower-income regions (*p* < 0.01), while it was not significant in high-income regions. It means that the increase of rural beds supply would reduce medical tourism of the rural population from the middle-low income regions, whereas, it has limited influence on the market concentration of urban inpatient visits in high-income regions.

## 4. Discussion

The results suggest two mechanisms for medical tourism among rural residents who utilize inpatient services of urban hospitals. First, the result of Table 2 shows that the rural old-age ratio had a significant and positive influence on aggregate inpatient visits of urban hospitals in the whole sample and subgroups. It means that the rural elderly had significant medical tourism towards urban hospitals in different income regions, respectively. The rural elderly from the high-income regions had more tendencies to resort to urban hospitals than those from the lower-income regions.

Second, we examined the influence of rural old-age ratio towards the market concentration of the inpatient visits from the urban hospitals. On average, it was found that the rise in rural old-age ratio would increase the market concentration of urban inpatient visits by 0.007. The rural old-age ratio significantly increased the market concentration of urban inpatient visits in middle-high income regions. It means that the rural elderly, especially from middle-high income regions, contributed to the market share of urban inpatient visits in the province. Medical tourism of the rural population was supported indirectly.

It was indicated that the urban old-age ratio was not significantly related to inpatient visits at urban hospitals. However, it was also found that the urban population size was positive and significantly related to the inpatient visits of urban hospitals. It could probably be due to the changes in life expectancy and the shift of disease patterns among the urban aged populations. Literature shows that with the improvement of living standards and life expectancy, the hospital need of the aged population would be reduced and chronic disease would increase [55,58]. Hence, the urban aged population had less probability to be in hospitals. At the same time, chronic diseases also spread widely in the working-age population [21,22,23]. Therefore, it is not surprising to find the positive coefficient of urban population size related to urban inpatient visits.

Other control variables also indirectly inferred the mobility of rural population for urban inpatient services. Literature supported that the improvement of drinking water would benefit the health of the rural population, hence reduced their medical need on certain conditions [49,50]. Results showed that the coverage of rural water improvement was negatively related to the urban inpatient visits and market concentration of urban inpatient visits. It is because the improvement of drinking water would benefit the health conditions of the rural population and reduce the need for urban inpatient services. Similarly, it was found that the urban road length was significant and positively related to the urban inpatient visits and its market concentration. The traffic facility showed an enabling factor for the mobility of the rural population for medical care. Finally, the rural beds’ facility would bring about rural urban hospital competition when there is a narrowing technological gap. It was negative and significantly related to the market concentration of urban inpatient visits. Hence it would modify the need for medical tourism from the rural population.

There are also limitations for this study. First, we limited the mobility of rural patients within the province and did not consider the inter-provincial medical seeking behavior. Second, the estimation bias may exist in the case we used an ecological approach to infer medical tourism from aggregate level data. Third, this study focused on the medical tourism caused by hospital needs and social class, not include individual perception and education factors. The above limitations are due to the availability of data. We would examine the mechanism of individual-level decision making process in future studies.

## 5. Conclusions

This study discusses the relationship between rural population aging and the urban hospital visits from an intra-province medical tourism framework. The research shows that the rural aged population had significant medical tourism to urban hospitals from different income groups. On average, a one percent increase in rural old-age ratio would improve the urban inpatient visits by 138 thousand persons (*p* < 0.01). The rural elderly from high-income groups had more tendencies towards medical tourism than the middle-low income groups.

The research also shows that the rural aged population—especially from the middle and high-income groups—had a positive and significant influence on the market concentration of urban inpatient visits in the province. On average, a one percent rise in the rural old-age ratio would increase the concentration of the urban inpatient visits by 0.007. Hence, medical tourism of rural population for urban inpatient services is indirectly supported.

The policy implications of the study are straightforward. Evidence of medical tourism of rural residents explains the differences in hospital utilization between urban and rural areas from a new routine. With the implementation of the intra-provincial referral systems, more attention must be paid to setting up branches of leading hospitals in suburban areas. When delivering exact medical resources, the central government must take into account not only the local urban population, but also the nearby rural population. The delivery of public health resources and facilities should be in a balanced way of fairness and efficiency. Except for the Chinese context, the result is also meaningful for the other developing countries with similar medical settings and fast urbanization. Therefore, the policy implications are worth using for their references.

## Figures and Tables

**Table 1:** Description of main variables.

Variables	Whole Sample	Low **Income**	Middle Income n = 198 Mean(SD)	High Income n = 55 Mean(SD)
	n = 319	n = 66		
	Mean(SD)	Mean(SD)		
Total inpatient visits to urban hospitals	242.4 (200.2)	111.723 (91.80)	246.62 (168.50)	383.86 (284.54)
HHI of urban inpatient visits	0.22 (0.16)	0.13 (0.08)	0.20 (0.13)	0.38 (0.21)
Explanatory variables:				
Rural old-age ratio	9.36 (2.20)	7.82 (1.62)	9.56 (2.18)	10.48 (1.94)
Urban old-age ratio	8.79 (1.98)	8.72 (2.04)	8.99 (1.74)	8.18 (2.55)
Rural population size	3183.62 (1985.97)	2095.1 (1331.82)	3461.57 (2096.00)	3489.27 (1797.58)
Urban population size	1287.19 (990.06)	497.27 (300.33)	1284.63 (679.80)	2244.32 (1510.73)
Rural per capita income	6795.53 (2978.51)	4516.34 (1549.57)	6484.89 (2077.33)	11000.00 (3432.84)
Urban per capita income	19,000.00 (5585.50)	16,000.0 (3892.70)	18,000.0 (4538.57)	25,000.00 (5600.00)
HHI of urban population	0.09 (0.07)	0.04 (0.02)	0.09 (0.06)	0.16 (0.08)
No. of beds per 100 rural persons	0.30 (0.10)	0.31 (0.10)	0.30 (0.10)	0.29 (0.11)
Ratio of rural water improvement	72.76 (20.95)	55.8 (26.69)	72.72 (15.76)	93.25 (6.45)
Urban road per capita	14.63 (3.93)	12.60 (3.21)	14.84 (3.89)	16.29 (3.89)
Urban road total length	11,000 (9941.08)	3217.83 (2152.96)	10000.0 (7505.53)	21,000.00 (14000.00)

Note: the unit of total urban inpatient and rural, urban population are 10,000 persons. The unit of rural and urban income is RMB Yuan. The unit of rural and urban old-age ratio is a percentage. The unit of rural water improvement is a percentage. The unit of urban road per capita is squared meters and the total length is kilometers. Since the acceptance of New Rural Cooperative schemes in the urban system normally after the year 2014, the factor of new rural cooperative schemes is not included.

**Table 2:** Rural old-age ratio and inpatient visits to the urban hospitals.

Column	(1)	(2)	(3)	(4)
VARIABLES	Whole sample	Low income	Middle income	High income
Rural old-age ratio	13.8546 ***	9.8501 **	9.4859 ***	23.0236 ***
(lagged)	(2.9218)	(4.9354)	(3.3429)	(8.7183)
Rural pop size	0.0610 **	–0.0569	–0.0926 ***	0.4098 ***
(lagged)	(0.0270)	(0.0561)	(0.0288)	(0.0284)
Urban old-age ratio	–0.4892	1.0131	–0.9211	–19.3115**
(lagged)	(1.5256)	(1.2210)	(1.8979)	(7.5315)
Urban pop. size	0.2814 ***	–0.1169	0.1758 ***	0.4842 ***
(lagged)	(0.0391)	(0.0792)	(0.0408)	(0.0420)
Rural per capita income	–0.0000	0.0068	0.0259 ***	–0.0831 ***
	(0.0050)	(0.0152)	(0.0097)	(0.0148)
Urban per capita income	0.0002	0.0191 ***	-0.0001	0.0639 ***
	(0.0009)	(0.0052)	(0.0008)	(0.0109)
Rural water	–1.0554 ***	–0.9739 **	–3.7604 ***	–11.0880 ***
Improvement	(0.3889)	(0.4079)	(0.4140)	(3.0258)
Urban total road	0.0187 ***	0.0462 ***	0.0152 ***	0.0001
Length	(0.0023)	(0.0058)	(0.0032)	(0.0028)
Beds per 100	–103.5130	211.0686 *	81.1574	–365.1136 *
Rural persons	(70.3831)	(115.1104)	(80.0862)	(212.7333)
Constant	–300.7799 ***	–89.7935	343.6834	193.1557
	(67.3026)	(209.6316)	(217.2397)	(419.4906)
Fixed Effect	YES	YES	YES	YES
Time Effect	YES	YES	YES	YES
Observations	319	66	198	55

Note: *** *p* <0.01, ** *p* <0.05, * *p* <0.1; values in parentheses indicate standard errors. Model was estimated by FGLS estimation.

**Table 3:** Rural old-age ratio and the concentration of urban inpatient visits (HHI).

Column	(1)	(2)	(3)	(4)
VARIABLES	Whole sample	Low income	Middle income	High income
Rural old-age ratio	0.0071 ***	–0.0010	0.0084 **	0.0295 **
(lagged)	(0.0028)	(0.0051)	(0.0037)	(0.0120)
HHI of urban	0.5508 ***	2.0938 ***	0.8174 ***	0.4603 *
population	(0.1375)	(0.3537)	(0.2226)	(0.2774)
Rural per capita income	0.0000 ***	–0.0000	0.0000	0.0000 *
	(0.0000)	(0.0000)	(0.0000)	(0.0000)
Urban per capita income	–0.0000	0.0000**	0.0000	–0.0000 **
	(0.0000)	(0.0000)	(0.0000)	(0.0000)
Urban old-age ratio	–0.0054 ***	0.0012	–0.0048 ***	–0.0185 **
(lagged)	(0.0014)	(0.0014)	(0.0018)	(0.0091)
Urban Road	0.0021	0.0076 ***	0.0031 *	0.0050
Per capita	(0.0013)	(0.0018)	(0.0016)	(0.0073)
Rural water	–0.0009 ***	–0.0004	–0.0010 **	–0.0014
Improvement	(0.0003)	(0.0005)	(0.0004)	(0.0035)
Beds per 100	–0.2297 ***	–0.3388 **	–0.2217 ***	–0.1049
Rural persons	(0.0643)	(0.1445)	(0.0786)	(0.2691)
Constant	0.4023 ***	–0.1387 *	0.0242	0.7037
	(0.0605)	(0.0824)	(0.0520)	(0.5572)
Fixed effect	Yes	Yes	Yes	Yes
Time effect	Yes	Yes	Yes	Yes
Observations	319	66	198	55

Note: *** *p* <0.01, ** *p* <0.05, * *p* <0.1; values in parentheses indicate standard errors. The model was estimated by FGLS estimation. The HHI of urban inpatient visits was recorded in a decimal.
